# Synergistic Analysis of Circulating Tumor Cells Reveals Prognostic Signatures in Pilot Study of Treatment-Naïve Metastatic Pancreatic Cancer Patients

**DOI:** 10.3390/biomedicines10010146

**Published:** 2022-01-11

**Authors:** Sarah Owen, Emily Prantzalos, Valerie Gunchick, Vaibhav Sahai, Sunitha Nagrath

**Affiliations:** 1 Department of Chemical Engineering, University of Michigan, Ann Arbor, MI 48109, USA; snowen@umich.edu (S.O.); eprantza@umich.edu (E.P.); 2 Biointerfaces Institute, University of Michigan, Ann Arbor, MI 48109, USA; 3 Division of Hematology and Oncology, Department of Internal Medicine, University of Michigan, Ann Arbor, MI 48109, USA; vagunchi@med.umich.edu; 4 Rogel Cancer Center, University of Michigan, Ann Arbor, MI 48109, USA

**Keywords:** CTC, liquid biopsy, pancreatic cancer, mutant KRAS, gene expression profiling

## Abstract

**Simple Summary:**

Pancreatic cancer is one of the most deadly cancer types because it usually is not diagnosed until the cancer has spread throughout the body. In this study, we isolate cancer cells found in the blood of pancreatic cancer patients called circulating tumor cells (CTCs) to study their mutation and gene expression profiles. Comparing patients with better and worse survival duration revealed signatures found in these cancer cells. Characterizing these signatures may help improve patient care by using alternative treatment options.

**Abstract:**

Pancreatic ductal adenocarcinoma is typically diagnosed at late stages and has one of the lowest five-year survival rates of all malignancies. In this pilot study, we identify signatures related to survival and treatment response found in circulating tumor cells (CTCs). Patients with poor survival had increased mutant KRAS expression and deregulation of connected pathways such as PI3K-AKT and MAPK signaling. Further, in a subset of these patients, expression patterns of gemcitabine resistance mechanisms were observed, even prior to initiating treatment. This work highlights the need for identifying patients with these resistance profiles and designing treatment regimens to circumvent these mechanisms.

## 1. Introduction

Pancreatic ductal adenocarcinoma (PDAC) remains one of the most deadly cancer types, with a five-year survival rate of less than 3% in patients with advanced disease [1]. Despite significant increases in survival rates of patients with other cancers, largely through early detection and the advent of targeted therapy, there has been only modest advancement in pancreatic cancer [2]. Pancreatic cancer still suffers from late diagnosis, due to the lack of available early screening methods, and absence of symptoms at early stages [3,4,5,6]. Additionally, current pancreatic cancer treatment options are mostly limited to chemotherapy, radiation, and surgery, or a combination of these approaches, with the exception of targeted and immunotherapies therapies in small number of patients with uncommon molecular alterations. The standard cytotoxic chemotherapy has modest clinical benefit along with a decrease in quality of life from potential side effects [7]. Patients with localized disease may be candidates for surgery, occasionally following upfront cytotoxic chemotherapy [7].

Due to the location of the pancreas within the body, tumor biopsies are particularly challenging [8]. Easily accessible biomarkers, such as from a liquid biopsy, are not approved for confirmation of diagnosis of PDAC but can be critical to aid in detection as well as monitoring [4]. Currently, the standard clinical biomarkers include carcinoembryonic antigen (CEA) and carbohydrate antigen 19-9 (CA19-9). In cohort studies, CA19-9 has been shown to correlate with the presence and stage of pancreatic cancer [9]; however, these can suffer from low sensitivity coupled with high false-negative rates [10,11] as well as a lack of specificity for pancreatic cancer [5].

Circulating tumor cells (CTCs) offer the potential to noninvasively access tumor cells through a routine blood draw. CTCs are shed from the primary tumor into the blood and may lead to the development of metastatic tumors. Despite their rarity in the blood, CTC abundance and phenotype have been shown to be independent prognostic factors for patient outcome in many cancer types [12,13,14,15,16,17,18]. However, further characterization of these CTCs may allow for the detection of aggressive genotypes and phenotypes and improve patient stratification [8,19,20].

Epithelial to mesenchymal transition (EMT) is one of the first steps in the metastatic cascade. EMT is implicated in tumor metastasis and drug resistance [21,22,23,24,25]. Recent studies have shown that CTC phenotypes may provide prognostic data in PDAC. The detection of vimentin, a mesenchymal marker, in CTCs showed an increased risk of recurrence, whereas the presence of epithelial CTCs was associated with worse overall survival [19]. Understanding the cellular regulation involved in EMT may present a way to improve cancer treatment.

Additionally, oncogenic KRAS is known to be mutated in greater than 90% of PDAC patients [26]. Mutated KRAS aids in the cellular transformation to aberrant cancer, uncontrolled cell growth, and promotes an invasive cell phenotype [27]. Additionally, KRAS mutations contribute to resistance to targeted therapies such as the epidermal growth factor receptor (EGFR) tyrosine kinase inhibitor (TKI) erlotinib [27,28]. While KRAS mutations have been shown to be detected in PDAC CTCs [29,30,31,32], the impact of KRAS mutations on CTC biology has not been studied.

**Figure 1:**
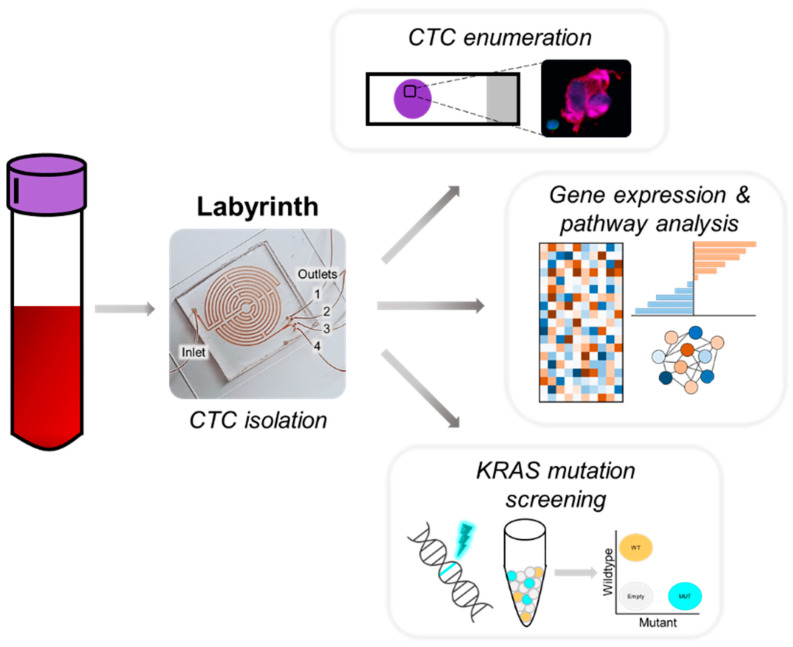
Multipronged CTC enumeration and transcriptome analysis from metastatic PDAC patients.

In this proof of concept, retrospective study, we characterize CTCs from metastatic, treatment-naïve PDAC patients. CTCs are enumerated and their EMT phenotype is characterized. A second, follow-up sample was obtained from patients after the initiation of therapy, and changes in CTCs were compared. Additionally, we evaluate the presence of KRAS mutations in CTCs and its expression before and during therapy. We performed comprehensive CTC transcriptome analysis, to identify prognostic gene expression signatures in CTCs informative of patient survival, and discuss novel molecular prognostic signatures (Figure 1) [8,29].

CTCs are isolated from the blood of metastatic PDAC patients using Labyrinth for label-free, sized-based isolation. The CTCs are enumerated and phenotyped using ICC. CTC transcriptomes and mutant KRAS expression are profiled using microarray and digital PCR, respectively.

## 2. Materials and Methods

### 2.1. Cell Culture

All cell lines were maintained at 37 °C, 5% CO_2_ under normoxic conditions. Panc-1 cells were grown in Dulbecco’s Modified Eagle’s Medium (DMEM) (Gibco, Waltham, MA, USA) supplemented with 10% fetal bovine serum (FBS) (Sigma, St. Louis, MO, USA), and 1% antibiotic-antimycotic (anti-anti) (Gibco, Waltham, MA, USA). MIA PaCa-2 were grown in DMEM supplemented with 10% FBS, 2.5% horse serum and 1% anti-anti. AsPC-1, and H1650 were grown in RMPI-1640 (Gibco, Waltham, MA, USA) supplemented with 10% FBS and 1% anti-anti. Cells were grown to 70–80% confluence before sub-culturing using 0.05% Trypsin-EDTA (Gibco, Waltham, MA, USA). Between cell passages, medium was replaced every 48–72 h. All cell lines were tested and reported negative for mycoplasma using MycoAlert^TM^ Mycoplasma Detection Kit (Lonza, Basel, Switzerland).

### 2.2. Labyrinth Fabrication

Labyrinths were fabricated using silicon wafer molds using soft lithography. Polydimethylsiloxane (PDMS) is prepared by prepared by mixing the elastomer base with a cross-linking agent at a ratio of 10:1 (Ellsworth Adhesive, Germantown, WI, USA). The mixture is poured onto the molds, degassed in a desiccator for 1–2 h, and cured at 75 °C overnight. The hardened PDMS is cut from the mold using a razor blade and inlet (1) and outlet (4) holes are punched through the PDMS using a 0.75 mm biopsy punch. The PDMS is bonded to a 2″ × 3″ glass slide via plasma bonding. After visual inspection for flaws under a microscope, the labyrinth is tubed with 10″ strands of 0.030″ outer diameter/0.010″ inner diameter Tygon^®^ tubing (Cole-Parmer, Veron Hills, IL, USA). The tubing is cut at a 45° angle and placed so that it does not impact the flow of device.

### 2.3. Patient Enrollment

The patients were enrolled in the Assessment of Biological Markers in Pancreatic Cancer study (HUM00025339). The study protocol was reviewed and approved by the University of Michigan Medicine Institutional Review Board (IRB). All patients gave informed consent to participate in this research study. Patients were enrolled based on the following criteria. Patients had metastatic PDAC and were treatment naive at the time of initial blood draw.

### 2.4. Sample Collection and CTC Isolation

Blood was collected into EDTA tubes and processed within 2 h of sample collection. Blood was processed as previously described [33]. In brief, blood volume was measured and divided into conicals. Red blood cells (RBCs) depletion was performed using HetaSep^TM^ (STEMCELL Technologies, Vancouver, BC, Canada), centrifuged at 90× *g* for 1 min at room temperature, with the centrifuge brake off. After centrifugation, the sample incubated at room temperature for an additional 10 min to allow further RBC sedimentation. The supernatant, containing nucleated cells, was collected and diluted with phosphate buffered saline (PBS) pH 7.4 (Gibco, Waltham, MA, USA) to 3× the original blood volume. The sample was processed through Labyrinth at a processing rate of 2500 µL/min. Before sample collection, flow was stabilized for 1 mL of flow, then the CTC outlet (Outlet 2) was collected in a separate conical. The final sample was divided for CTC enumeration and transcriptomic analysis.

### 2.5. Immunocytochemistry and CTC Enumeration

A portion of the CTC-enriched sample after Labyrinth processing was used for immunofluorescence. Polysine^TM^ microscope slides (Thermo Scientific, Waltham, MA, USA) were prepared in EZ Cytofunnel (Thermo scientific) and each loaded with the 200 µL of CTC sample. The samples were spun 800 rpm for 10 min at room temperature. The cells were fixed using 200 μL of 4% paraformaldehyde (PFA), which was added to the Cytofunnel and spun again using the same conditions. Slides were covered in PBS and stored at 4 °C for up to 2 weeks before immunofluorescent staining.

Slides were permeabilized with 0.2% Triton X-100 for 3 min at room temperature, washed 3× with PBS, then blocked with 10% goat serum (Life Technologies, Carlsbad, CA, USA) for 30 min at room temperature. Slides were incubated with primary antibodies (Table 1) diluted in 10% goat serum overnight at 4 °C. The following day, slides were washed 3× using PBS, each with a 5 min incubation. Slides were incubated, in the dark with secondary antibodies (Table 1) diluted in 10% goat serum for 45 min at room temperature. The slides were then washed 3× using PBS, each with a 5 min incubation. Coverglass was mounted using Prolong Gold Antifade Mountant with DAPI (Invitrogen, Waltham, MA, USA).

Slides were imaged using Nikon TI microscope at 20× magnification. The entire sample area was scanned and the resulting tiled images were manually analyzed. CTCs were identified based on the following criteria. CTCs are defined as DAPI+/CD45−/CK+. The CTC phenotype was further characterized based on vimentin protein expression. Ep-CTCs are defined as DAPI+/CD45−/CK+/VIM− and EMT-CTCs are defined as DAPI+/CD45−/CK/VIM+.

The enumerated CTCs on the slides were used to calculate the CTCs/mL and are reported as per mL of whole blood. Each slide was loaded with 200 µL of post-Labyrinth CTC product. The recovered CTCs/mL of blood processed was calculated using the CTCs/slide and CTC product volume normalized to original sample blood volume.

### 2.6. RNA Extraction

A portion of the sample was used for transcriptomic analysis. The CTC-enriched sample was centrifuged at 300× *g* for 10 min to pellet the cells. The cell pellet was lysed in 700 µL of TRIzol^TM^ Reagent (Life Technologies, Carlsbad, CA, USA) and incubated for 5 min at room temperature. The lysed sample was immediately frozen at −20 °C for up to 2 weeks before RNA isolation and purification.

RNA was purified using an in-house, modified protocol using the Total RNA Purification kit (Norgen Biotek Corp., Thorolod, ON, CA). The protocol utilized TRIzol^TM^ Reagent to lyse the sample to increase RNA yield in the presence of the remaining blood components, mainly RBCs. After the sample in TRIzol^TM^ Reagent was thawed, 140 µL of chloroform was added and vortexed to mix. After 3 min of incubation at room temperature, the sample was centrifuged at 12,000× *g* for 15 min. The aqueous layer (RNA layer) was collected and mixed with equal volume 70% ethanol and added to the provided columns (Norgen Biotek Corp., Thorolod, ON, CA). The columns were washed 3× with the provided Wash Solution A (Norgen Biotek Corp., Thorolod, ON, CA), and eluted into 30 µL of provided Elution Solution A (Norgen Biotek Corp., Thorolod, ON, CA).

All purified RNA, cDNA, and PCR reagents were handled in a PCR workstation to prevent nuclease contamination.

### 2.7. cDNA Synthesis, and Droplet Digital PCR (ddPCR)

cDNA was prepared using SuperScript IV VILO with ezDNase^TM^ Enzyme (Invitrogen, Waltham, MA, USA) following the manufacturer’s protocol.

The 20× ddPCR KRAS G12/G13 Screening Multiplex Assay (Bio-Rad Laboratories, Hercules, CA, USA) was used with a modified protocol. 25 µL reactions were prepared combining (1) the provided master mix, (2) the primer/probes, and (3) the cDNA sample. A modified primer/probe concentration was optimized yielding a reduced primer concentration of 432 nM, and probe concentration of 120 nM. CTC samples were analyzed for mutation status using the RainDance RainDrop ddPCR system. cDNA was partitioned into aqueous reaction droplets. Analysis was performed using the RainDrop Analyst II Software (RainDance Technologies, Lexington, MA, USA). Gating templates were generated using positive and negative cell line controls.

### 2.8. Affymetrix Microarray Processing and Data Analysis

Total RNA was processed using Affymetrix GeneChip^®^ WT PLUS Expression (Affymetrix, Santa Clara, CA, USA) Arrays. Total RNA concentration was evaluated by nanodrop spectrophotometer and processed either at the University of Michigan Advanced Genomics core or at the Affymetrix Bioinformatics Services core (at Thermo Fisher) following standard protocol. Data were returned and processed using Transcriptome Analysis Console (TAC) software (Thermo Fisher, Waltham, MA, USA). Data were normalized for batch effects. All analysis was performed using the criteria of a fold change >2 or <−2 and *p*-value < 0.05 between the two specified groups.

### 2.9. Statistical Analysis

Statistical analysis was performed using GraphPad Prism 8.4.2 (GraphPad, San Diego, CA, USA). Significance and *p*-values for non-matched samples were determined using a two-sided unpaired t-tests assuming a Gaussian distribution, with a 95% confidence level. Significance and p-values for matched samples (pre-treatment vs. on-treatment) were determined using a two-sided paired t-tests assuming a Gaussian distribution, with a 95% confidence level. Plotted error bars represent the standard deviation.

## 3. Results

### 3.1. Patient Characteristics

Fifteen patients provided informed consent under an institutional approved IRB protocol. All patients were diagnosed with metastatic, treatment-naïve PDAC (Table 2). Blood samples were collected prior to the start of therapy from 15 patients, and then again in 10 of these patients after therapy had begun (*n* = 25 total samples). The mean time the patient was on therapy prior to follow-up sample collection was 23 days. Patients were evenly split between male and female, with a median age of 67 years (range: 48–85 years). Full patient demographics are shown in Table S1.

### 3.2. Detection of CTCs for Phenotypic and Molecular Profiling

CTCs were isolated from the blood using the Labyrinth, an inertial microfluidic technology that isolates CTCs based on size with >90% recovery across various solid tumor types and depletes > 90% of white blood cells [31,32,33] (Figure 1). The isolated CTCs were split across three analyses. A small fraction (~5%) was used for CTC enumeration using immunocytochemistry (ICC). The remaining sample was divided for KRAS mutation using droplet digital PCR (ddPCR) and gene expression profiling using GeneChip^®^ Expression Arrays.

However, due to sample availability, CTC enumeration was performed for 14/15 pre-treatment samples and 8/10 on-treatment samples. Transcriptome profiling was performed on 10 pre-treatment and 9 on-treatment samples. KRAS mutational profiling was performed on 13 pre-treatment and 10 on-treatment samples.

### 3.3. CTCs Display Heterogeneous Abundance, EMT Phenotype, and KRAS Mutation Burden

**Figure 2:**
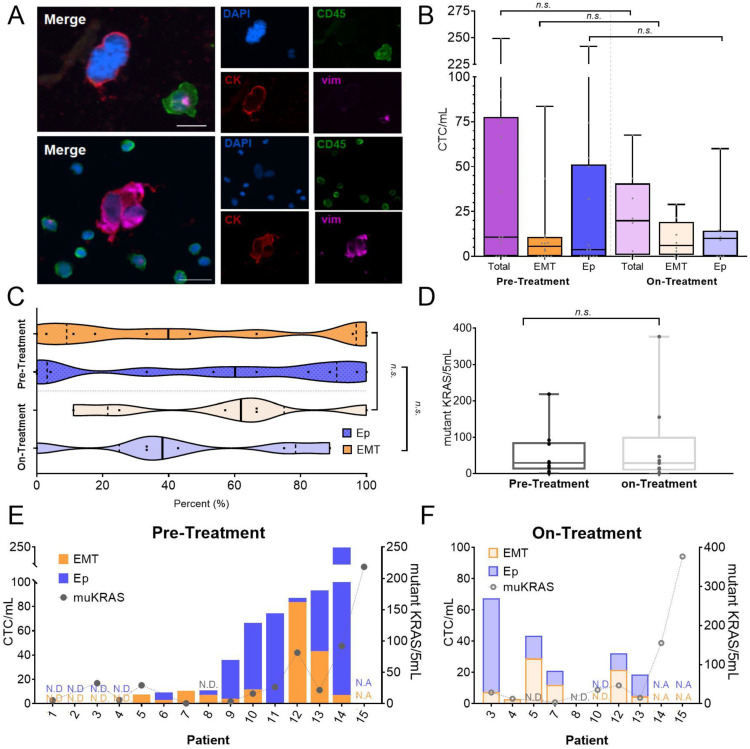
CTC enumeration, phenotype and mutant KRAS burden profiling. (**A**) Image of an Ep CTC (top panel) and two EMT CTCs (bottom panel) surrounded by white blood cells. Scale bar represents 20 µm. (**B**) CTC enumeration based on phenotype for pre-treatment and on-treatment samples. (**C**) Distribution of CTC phenotype based on time point. (**D**) Mutant KRAS expression assessed by ddPCR in CTCs based on time point. (**E**,**F**) Comparison of CTC counts based on phenotype with mutant KRAS (muKRAS) expression for (**E**) pre-treatment and (**F**) on-treatment CTC samples. N.A. = not available. N.D. = not detected. *n.s.* = not significant.

CTCs were identified based on the expression of cytokeratin (CK), a marker of epithelial origin, and absence of CD45, a leukocyte marker (Table 1). Phenotype was determined based on the expression of vimentin in EMT-like CTCs (EMT-CTCs), while epithelial-like CTCs (Ep-CTCs) were vimentin negative (Figure 2A). CTCs were detected in 16/22 (73%) of samples. Across all patients and time points, the median CTC burden in patients detected by ICC was 14.79 CTCs/mL blood (range: 0–249 CTCs/mL blood.)

Based on treatment status, CTCs were detected in 10/14 (71%) of pre-treatment samples, ranging from 0 CTCs/mL to 249 CTCs/mL blood, with an average of 46.1 ± 65.6 total CTCs/mL (Figure 2B). While nearly all patients with CTCs had both EMT-CTC and Ep-CTC populations, patients displayed the entire spectrum of proportions of EMT- and Ep-CTC populations. Of the 10 patients with detectable CTCs, seven had both Ep-CTCs and EMT-CTCs (70%), two patients having only EMT-CTCs and one patient having only Ep-CTCs. However, the relative abundance of these two CTC subpopulations, was highly patient dependent, ranging from 3 to 96% (average: 45.7 ± 38.6%) EMT-CTCs in patients with both phenotypes (Figure 2B,C).

CTC detection rates by ICC of on-treatment samples were similar to pre-treatment samples; CTCs were detected in in 6/8 (75%) of on-treatment samples compared to 10/14 (71%) of pre-treatment samples. However, CTC levels tended to be lower during treatment, compared to pre-treatment samples. CTCs ranged from 0 to 68 CTCs/mL, with an average of 23.2 ± 22.3 CTCs/mL (Figure 2B). Despite lower total CTCs, there was a shift towards a higher percentage of EMT-CTCs, similar to what we have previously reported [31] (Figure 2C). On-treatment samples showed an average of 58.1 ± 33.8% EMT-CTCs, nearly a 30% increase compared to pre-treatment CTC samples. One patient had entirely EMT-CTCs, while the other five patients with detected CTCs had both EMT-CTCs and Ep-CTCs, ranging from 43 to 67%. At both time points, there was not an observed correlation between the total CTC abundance and proportion of a CTC phenotype subpopulation.

CTC samples were further tested for the presence and abundance of KRAS G12/13 mutations. RNA extracted from the CTCs was analyzed for KRAS mutations (muKRAS) using ddPCR. ddPCR is an ultrasensitive technique enabling single molecule detection, critical in low input samples [34]. The ddPCR system was optimized and validated for use with a KRAS mutation detection assay for CTCs, which simultaneously screens for the 7 most frequent clinically detected mutations in the G12/G13 region of the KRAS gene (Figure S1). Mutant KRAS was detected in 12/13 (92%) of pre-treatment CTC samples. Patients with KRAS mutations had an average of 48.0 ± 59.5 mutant KRAS transcripts/5 mL blood (Figure 2D). Mutant KRAS was detected in 8/10 (80%) of on-treatment CTC samples with an average of 84.3 ± 119.4 mutant KRAS transcripts/5 mL blood, despite overall lower CTCs (Figure 2D). These detection rates are in agreement with reported literature that 90% of PDACs contain a KRAS mutation [26]. Previous studies in CTCs for KRAS mutation have primarily focused on evaluating KRAS mutations at the genetic level and only at a single time point [29,30,35,36]; however, the level of mutant KRAS expression in CTCs may be influenced by treatment status (Figure 2D). With the exception of patient 5, all patients maintained their KRAS mutation status despite changes in mutant KRAS expression across time points. Patient 8 had no detectable mutant KRAS at both time points, and the remaining patients were positive at both time points.

In most patients, the abundance of mutant KRAS expression trended with the number of EMT-CTCs, observed most prominently in pre-treatment CTC samples, but this trend was still mildly observed in on-treatment samples (Figure 2E,F). However, since only a small fraction of the sample was used for CTC enumeration, while the rest was dedicated for molecular characterization, it is possible there was sampling bias during the enumeration, which would account for the mutant KRAS detected in samples with no CTCs detected based on enumeration.

### 3.4. Monitoring Treatment-Related CTC Dynamics

**Figure 3:**
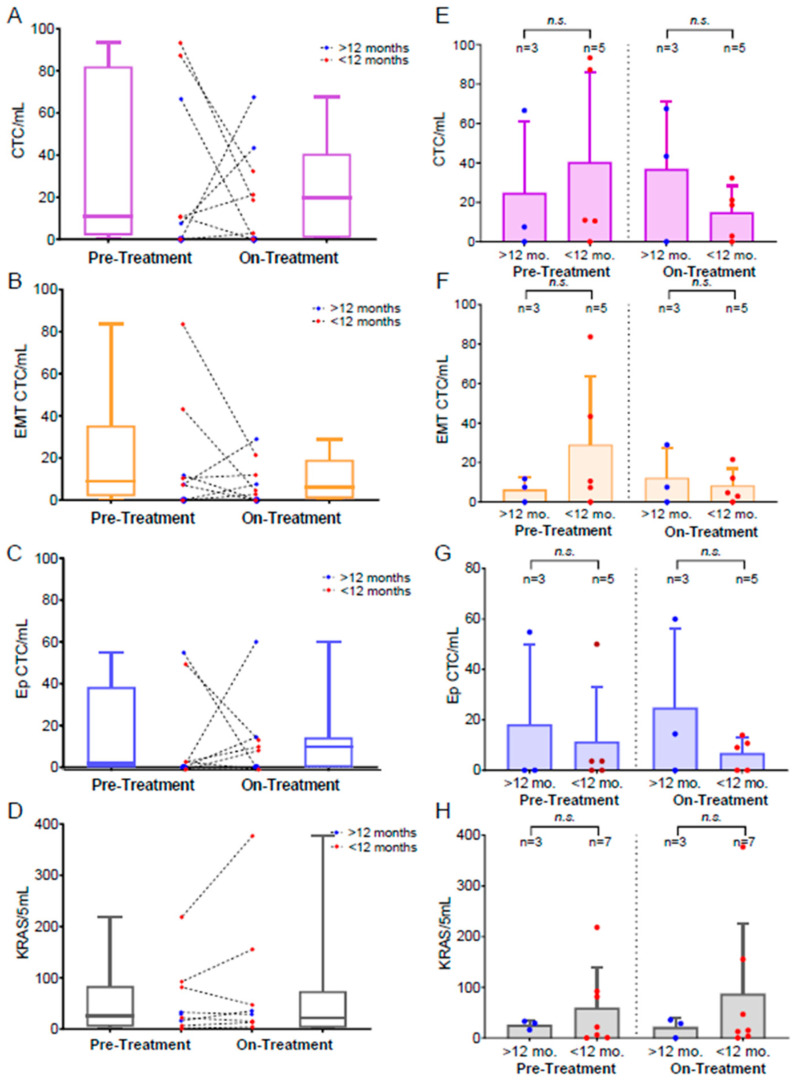
CTC dynamics based on patient survival in matched patients. (**A**–**D**) Distribution of pre-treatment and on-treatment CTC samples with individual patient trajectories mapped for (**A**) total CTCs (*n* = 8 patients), (**B**) EMT-CTCs (*n* = 8 patients), (**C**) Ep-CTCs (*n* = 8 patients), and (**D**) mutant KRAS expression (*n* = 10 patients). Patients with survival of <12 months are shown with red dots and patients with survival of >12 months are shown with blue dots. (**E**–**H**) Comparison based on patient survival for (**E**) total CTCs, (**F**) EMT CTCs, (**G**) Ep CTCs, and (**H**) mutant KRAS. Error bars represent the standard deviation. *n.s.* = not significant.

Ten patients had matched pre-treatment and on-treatment CTC samples. Of these ten, all showed KRAS mutation profiling and eight had quantified CTCs. The matched pre-treatment and on-treatment samples were compared for each patient. The CTC burden in response to therapy showed significant variability between patients, with four patients having increased CTCs (patients 3–5, 7), and four with decreased CTCs once receiving therapy compared to pre-treatment CTC levels (patients 8,10,12,13) (Figure 3A). Interestingly, of the four patients who had increased total CTC counts, three had increases in both EMT-CTCs and Ep-CTCs, with one patient showing an increase in only EMT-CTCs. Strikingly, all four of these patients had the lowest pre-treatment CTC abundance. Similarly, the four patients with decreased CTCs, all showed a decrease in EMT-CTCs and with three of the four patients also having decreased Ep-CTCs, while only one had an increase (Figure 3B,C). Of the nine patients who had detectable mutant KRAS levels from at least one time point, five had increased levels after beginning therapy, while four had decreased, one of which completely lost mutant KRAS signal (Figure 3D).

To evaluate the clinical relevance of these dynamics, the patients with samples analyzed at both time points were stratified based on patient survival duration, either greater or less than 12 months after initiating therapy (Table 2). While each patient demonstrated a unique trajectory (Figure 3A–D) there was a mild trend that patients with poor survival demonstrated a higher total CTC burden (Figure 3E) as well as higher EMT-CTCs. Ep-CTCs did not show a correlation with patient survival duration (Figure 3G). Mutant KRAS expression levels tended to be higher at both time points in patients with poor survival compared to those with extended survival; however, it was not statistically significant, which could be due to the small size of this pilot cohort (Figure 3H).

**Figure 4:**
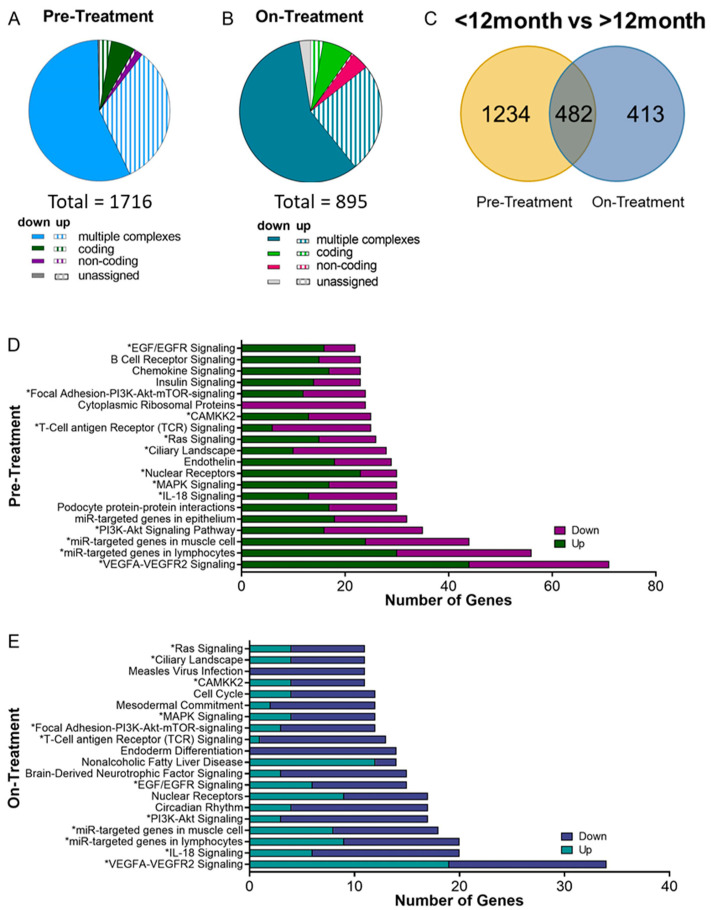
CTC differential gene expression and pathway analysis based on patient survival. (**A**,**B**) Differential gene expression based on patient survival duration for (**A**) pre-treatment and (**B**) on-treatment CTC samples. (**C**) Venn diagram comparison of differentially expressed genes based on survival at time points, pre-treatment only, on-treatment only, or both pre- and on-treatment. (**D**,**E**) Top 20 deregulated pathways based on number of differentially expressed genes in pathway between patients with survival <12 months and survival >12 months for (**D**) pre-treatment and (**E**) on-treatment CTC samples. * indicates statistically significant pathway deregulation between poor and improved treatment (*p* < 0.05).

The CTC samples were further profiled for differentially expressed genes and deregulated pathways based on patient survival duration. Total RNA was exacted from the CTC samples and processed using Affymetrix GeneChip^®^ Expression Arrays for whole-transcriptome profiling. To compare patient groups, differential expression was defined as fold change > |2| and *p* < 0.05 [37] and analyzed using the Transcript Analysis Console (TAC) software (Thermo Fisher, Waltham, MA, USA). In the pre-treatment samples, there were 1716 genes that were significantly differentially expressed between patients with poor survival compared to those with extended survival (Figure 4A), compared to only 895 in the on-treatment samples (Figure 4B), with 482 of the genes being commonly differentially expressed across both time points (Figure 4C).

In pre-treatment samples, the fold change between differentially expressed genes in patients with <12 month survival versus >12 months survival ranged from 34.81 to −15.94, with some of the top differentially expressed genes being MMP9, Zeb2, S100P, MKI67, ALDHA2 and BCL2 (Table S2), which are known to be involved with cell phenotype, survival and proliferation, and disease aggressiveness [38,39,40]. Further, multiple S100 series A (S100A) genes, S100A8, S100A9, S100A11, and S100A12 were all upregulated in patients with poor survival (fold change, range = 4.73–34.81). S100A genes have shown growing interest in their involvement in pancreatic cancer progression, and in epithelial to mesenchymal transition [41,42]. In on-treatment samples, the differentially expressed genes had fold changes ranging from 35.55 to −11.46, and contained some overlapping top differentially expressed genes with pre-treatment samples, including as MKI67, ALDHA2 and A disintegrin and metalloproteinase 9 (ADAM9) (Table S2).

The ADAMs family of proteins is involved in several biological processes including the cell adhesion and migration, and proteolytic processing of other transmembrane proteins. ADAM9, has been previously reported to be upregulated in pancreatic cancer [43], and high ADAM9 expression in PDAC has been correlated with poor patient survival [44,45]. In these CTC samples, ADAM9 was shown to be upregulated at both time points in patients with poor survival. Further, the CXC chemokine, CXCL16 was shown to be upregulated only in pre-treatment CTC samples of patients with survival <12 months. CXCL16 has been shown to have higher expression in PDAC compared to healthy tissue, and is readily cleaved by ADAM proteins. Soluble CXCL16 has been shown to increase the invasiveness of pancreatic cancer cells [46] and ovarian cancer [47].

Additionally, patients with reduced survival were found to have decreased MYC expression at both time points (Table S2). Increased MYC expression has been correlated with a more stem-like phenotype in other cancer types; however, Sancho et al. reported in pancreatic cancer that MYC expression was correlated with a differentiated phenotype [48].

Beyond individual differentially expressed genes, many pathways were shown to be deregulated based on patient survival duration. The list of differentially expressed genes based on patient survival of <12 months or >12 months identified above was mapped to their known pathways. Deregulated pathways were then identified using the number of differentially expressed genes involved in each pathway. Similarly, the extent of deregulation was ranked by the number of differentially expressed genes for each pathway; the top 20 most deregulated pathways based on survival are shown prior and during treatment (Figure 4D,E). Notably, known targets of oncogenic KRAS: mitogen-activated protein kinase (MAPK) signaling and phosphoinositide 3-kinase (PI3K)–AKT–mechanistic target of rapamycin (mTOR) signaling were shown to be differentially regulated based on patient survival duration [1]. Thirteen of the top 20 pathways appeared at both time points such as: VEGFA-VEGFR2, IL-18, Ras, PI3K-Akt and EGF/EGFR signaling. (Figure 4D,E, Table S3). Interestingly, it is to be noted that the differentially expressed genes within each pathway only had moderate overlap across the two time points (Table S3). For genes that were differentially expressed at both time points, in every case it was differentially expressed in the same direction (i.e., up at both time points, or down at both time points).

Beyond being deregulated across both time points, many of these pathways have been shown to be highly clinically relevant. VEGFA-VEGFR2 is known to increase the motility of pancreatic cancer cells and induce invasion, migration, and metastasis [49]. IL-18 has been shown to be increased in pancreatic cancer and can contributed to immunosuppression via regulatory B cells [50]. The role of oncogenic Ras activation in PDAC initiation and progression of PDAC has been well documented, with PDAC being considered one of the most RAS-addicted cancers [51]. PI3K/Akt signaling pathway regulates the proliferative, invasive, and metastatic behaviors of PDAC [52]. Together, these results demonstrate that the CTCs, shed from PDAC tumors, present in circulation represent a population that are enriched for invasive, proliferative, and highly motile characteristics, especially in the patients who had poor survival.

### 3.5. Evaluating the Influence of Treatment Type on CTC Dynamics

**Figure 5:**
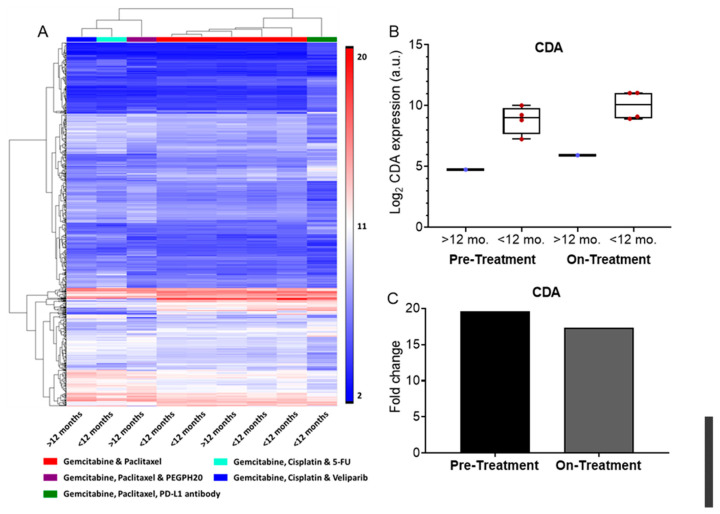
Effect of treatment on CTC transcriptome from on-treatment samples. (**A**) Hierarchical clustering of on-treatment CTC samples. Treatment is denoted with the colored bars at the top and patient status is shown below. (**B**) Log2 expression of CDA for patients received gemcitabine and paclitaxel therapy. (**C**) Fold change in expression of CDA based on survival <12 months and survival >12 moth at each time point for patients received gemcitabine and paclitaxel therapy.

Up to this point, all the samples have been stratified based on the time point or the patient survival duration, <12 months of >12 months. However, the patients in this cohort received diverse treatment regimens (Table 2). The findings thus far highlight that a pre-treatment, single time point assessment may be insufficient to broadly predict patient prognosis across different treatment plans, and a more tailored approach is necessary. Patients received one of five different treatments regimes. All patients were treated with the chemotherapeutic agent gemcitabine combined with at a minimum a second chemotherapeutic agent, either paclitaxel or cisplatin (Table 2). On-treatment CTC differential gene expression profiles were compared using unbiased hierarchical clustering. CTC samples clustered based on the treatment each patient was receiving (Figure 5A), with similar treatment types also clustering closely together. Patients receiving cisplatin were differentiated from those receiving paclitaxel, independent of the specific combination therapy the patient was receiving. Strikingly, the differences induced by these two chemotherapy agents was stronger than those caused by the other treatments, such as the poly ADP ribose polymerase (PARP) inhibitor veliparib, PD-L1 immunotherapies, or the hyaluronidase, PEGPH20.

In our cohort, only the gemcitabine and paclitaxel combination therapy had multiple patients, for whom we had CTCs transcriptome profiling. Therefore, only these patients were further evaluated. In this subset of patients there was a strong differential expression of cytidine deaminase (CDA) based on patient survival duration, with patients with <12 months survival having approximately 20 fold higher expression (Figure 5B,C). CDA is known to inactivate gemcitabine and can lead to drug resistance [53]. Interestingly, this differential expression was observed both prior to and while receiving therapy.

## 4. Discussion

Metastatic pancreatic cancer is extremely lethal, with a median survival of 12 months, and a five-year survival rate of less than 3% [1]. CTCs may provide a minimally invasive approach to predict patient response. In this study, we used a multipronged approach to evaluate CTC signatures of patients diagnosed with metastatic pancreatic cancer. CTCs were profiled based on their abundance, phenotype, KRAS mutation and transcript levels, and gene expression signatures. In this cohort of initially treatment-naïve patients, the number of EMT-CTCs prior to treatment showed a trend towards poorer survival. Furthermore, a shift towards more EMT phenotype during therapy was associated with poor survival.

We identified mutant KRAS in 20/23 (87%) of CTC samples. Previous CTC studies have identified KRAS mutant genotypes [29,30] but did not study the temporal changes with onset of therapy. Of the ten patients with matched pre- and on-treatment samples, we observed mutant KRAS in 9/10 patients with loss of KRAS mutation in the on-treatment sample for one patient. Interestingly, loss of KRAS was associated with a significant objective response compared to other patients. This patient is still alive 15 months after beginning therapy. Further, we observed increased mutant KRAS expression in patients with poor survival. This was associated with deregulation of known connected pathways such as PI3K-AKT-mTOR and MAPK signaling. Highlighting the need to not only screen for the presence, but also the expression levels of oncogenic mutations.

CTCs revealed treatment-specific gene expression profiles. These signatures highlight the utility of CTCs to monitor tumor cell changes over the course of therapy, which is not possible through a traditional biopsy due to the invasiveness of the procedure and location of the pancreas within the body, making it particularly challenging to access. In the subset of patients being treated with gemcitabine and paclitaxel, we observed higher expression of CDA, a known inactivator of gemcitabine, in patients with poor survival even prior to the beginning of therapy. Interestingly, this observation was conserved across the entire cohort in pre-treatment samples (survival <12 months vs. >12 months fold change = 22.5), but was lost when comparing on-treatment samples receiving different treatment plans. This suggests that the incorporation of other drugs into the treatment plan may circumvent CDA-mediated gemcitabine resistance, but additional work is needed to investigate this.

Effectively treating pancreatic cancer has been particularly challenging, believed largely to be due to the dense stroma. This desmoplasia limits the amount of cytotoxic agents that reach the tumor cells, therefore stoma modifying agents, such as PEGPH20, are being investigated to allow increased tumor penetration of the drug. However, phase III clinical trial evaluating PEGPH20 was recently stopped after showing no significant improvement compared to the control arm [54]. Interestingly, in our study, only one patient—patient 3, who was receiving PEGPH20—saw a large increase in CTCs on beginning treatment. Prior to treatment, no CTCs were detected. However, on beginning therapy, this number drastically rose to 67.5 CTC/mL. Further, nearly 90% of the CTCs were Ep-CTCs, the highest proportion of Ep-CTCs detected in on-treatment samples. It is possible that the use of PEGPH20, and therefore decreased stroma, allowed for extravasation of tumor cells into the bloodstream without undergoing EMT. While this is only one patient, it does raise the concern that improving tumor access for the drug may have unintended consequences such as easier tumor cell extravasation into the blood leading to increased CTCs and potentially driving metastasis. Further studies are needed to investigate the role of stroma modify agents on the presence of CTCs.

Collectively, we have shown that CTCs, despite their rarity in the blood, can enable multifunctional studies to evaluate signatures of patient survival in PDAC. While this work focused on CTC characterization, there are other rare cell types that have been reported in the blood. The multipronged analysis method presented in this manuscript can be applied to these other rare cell types, such as the tumor-macrophage fusion cells which display both epithelial and macrophage/myeloid markers. While these events have been reported in up to >40% of samples depending on the cancer type and enrichment method, the disease implications of these fusion cells are still under investigation [55]. However, early studies, including those in PDAC, have presented evidence, that similar to CTCs, they can contribute to the metastatic process and their abundance corelates with disease stage and patient survival [56,57,58,59]. Future studies are needed to directly analyze and compare CTCs with these other rare cell events.

## 5. Conclusions

This study isolated CTCs from fifteen metastatic, treatment-naïve pancreatic cancer patients using the microfluidic Labyrinth technology. Follow-up CTC samples were collected from 10/15 patients after the patient began therapy. CTC presence, abundance, and phenotype distribution varied between patients; however, EMT-CTCs tended to become a more predominate phenotype for patients receiving cancer therapy.

Further, while for the majority of patients mutant KRAS expression was detected in CTC samples, those with poor survival tended to have higher mutant KRAS expression at both time points compared to patients with increased survival. These patients were also found to have deregulated gene expression profiles in key pathways known to be involved in disease progression and spread.

Taken together, the work presented here showed mutant KRAS expression and transcriptome analysis carried mechanistic differences in CTCs between patients that corresponded to patient outcomes. This study also revealed considerations for personalized treatment design based on CTC gene expression signatures.

## Figures and Tables

**Table 1:** List of primary and the corresponding secondary antibodies used in the staining panel.

Primary Antibody	Catalog Number	Host (Isotype)	Secondary Antibody	Catalog Number	Host (Isotype)
Pan Cytokeratin	BioRad MCA1907	Mouse (IgG1)	Anti-Mouse IgG1, AF546	Thermo/Invitrogen A21123	Goat (IgG)
CD45	BioRad MCA87GA	Mouse (IgG2a)	Anti-Mouse IgG2a, AF488	Thermo/Invitrogen A21131	Goat (IgG)
Vimentin	Cell Signaling 5741	Rabbit (IgG)	Anti-rabbit IgG (H+L), AF647	Thermo/Invitrogen A21245	Goat (IgG)

**Table 2:** Patient Treatment and Clinical Status.

Patient	Visit	Treatment Status	All Treatments Used	Time on Treatment (Days)	Clinical Status	Time from Intervention Until Death (Days)
1	1	Treatment-naïve	Gemcitabine	-	Deceased	21
2	1	Treatment-naïve	Gemcitabine & Paclitaxel	-	Deceased	9
3	1	Treatment-naïve	Gemcitabine, Paclitaxel, & PEGPH20	-	Alive (587 days)	NA
	2	On-treatment		10		
4	1	Treatment-naïve	Gemcitabine & Paclitaxel	-	Deceased	132
	2	on-treatment		14		
5	1	Treatment-naïve	Gemcitabine & Paclitaxel; added Capecitabine after 242 days	-	Alive–Progression (476 days)	NA
	2	On-treatment		21		
6	1	Treatment-naïve	Gemcitabine & Paclitaxel	-	Deceased	24
7	1	Treatment-naïve	Gemcitabine & Paclitaxel	-	Deceased	123
	2	On-treatment		28		
8	1	Treatment-naïve	Gemcitabine, Paclitaxel & PD-L1 antibody	-	Deceased	100
	2	On-treatment		14		
9	1	Treatment-naïve	Gemcitabine & Paclitaxel	-	Deceased	433
10	1	Treatment-naïve	Gemcitabine, cisplatin & veliparib; after 370 days veliparib only	-	Alive–Continuing Treatment (593 days)	NA
	2	On-treatment		20		
11	1	Treatment-naïve	Gemcitabine; after 37 days Paclitaxel added	-	Deceased	138
12	1	Treatment-naïve	Gemcitabine, Paclitaxel & PD-L1 antibody	-	Deceased	212
	2	On-treatment		14		
13	1	Treatment-naïve	Gemcitabine & Paclitaxel	-	Deceased	147
	2	On-treatment		55		
14	1	Treatment-naïve	Gemcitabine & Paclitaxel	-	Deceased	42
	2	On-treatment		26		
15	1	Treatment-naïve	Gemcitabine, Cisplatin, 5-Fu; after 112 days Cisplatin replaced with Oxaliplatin; after 161 days Gemcitabine only	-	Deceased	317
	2	On-treatment		28		

## Data Availability

The data presented in this study are available on request from the corresponding author. The data are not publicly due to patient consent.

## Supplementary Materials

The following are available online at https://www.mdpi.com/article/10.3390/biomedicines10010146/s1, Figure S1: Optimization of KRAS G12/13 ddPCR assay; Table S1: Complete patient demographics; Table S2: Key differentially expressed genes in pre-treatment and on-treatment CTC samples based on patient survival duration; Table S3: Differentially expressed genes in thirteen overlapping deregulated pathways in pre-treatment and on-treatment samples.
