# Supplementary material for: Synergistic Analysis of Circulating Tumor Cells Reveals Prognostic Signatures in Pilot Study of Treatment-Naïve Metastatic Pancreatic Cancer Patients
Source: Biomedicines. 2022 Jan 11;10(1):146. doi: 10.3390/biomedicines10010146 (PMC8773204; doi:10.3390/biomedicines10010146)
Supplement: Supplementary file 1 [file biomedicines-10-00146-s001.zip › biomedicines-1482894-supplementary.pdf]

Supplementary Information

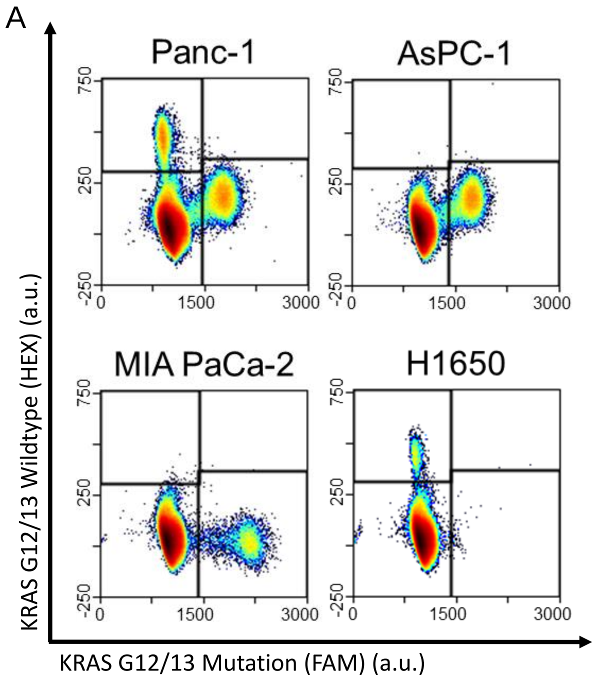

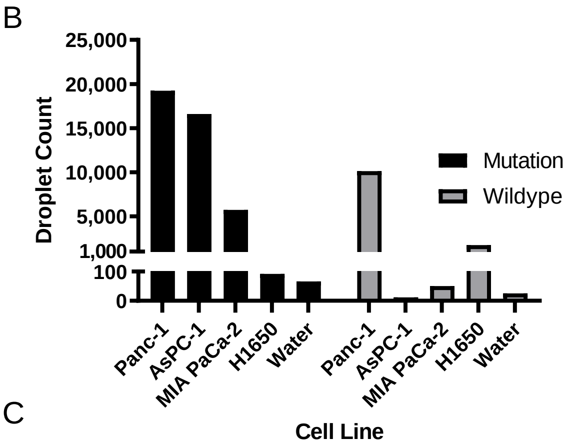

C

KRAS	Panc-1	AsPC-1	MIA-PaCa-2	H1650
Mutant	+	+	+	-
Wildtype	+	-	-	+
	Heterozygous	Homozygous	Homozygous	Homozygous

Figure S1: Optimization of KRAS G12/13 ddPCR assay.

(**A**) Control cell lines were used for KRAS ddPCR assay optimization. Panc-1, AsPC-1 and MIA Paca-2 were used as positive controls, while H1650 was used as a negative control. Due to noise in ddPCR results, a signal intensity threshold was set beyond the empty droplet population. Gates were drawn 100 intensity units away from the least dense layer of the empty droplet population to remove the background. (**B**) Raw droplet counts from 5ng of cDNA from the tested cell line samples. In patient samples droplet counts were normalized by subtracting our negative control sample droplet counts. (**C**) Table showing the observed KRAS mutation status of the four cell line control samples.

Table S1: Complete Patient Demographics

Patient	Diagnosis and Disease Stage	Sex	Race	Ethnicity	Age (years)	Alcohol Consumption	Pack-Years Smoking
1	metastatic	M	White or Caucasian	Non-Hispanic	68	N	11
2	metastatic	F	White or Caucasian	Non-Hispanic	54	Y	2.5
3	metastatic	F	White or Caucasian	Non-Hispanic	67	N	3.75
4	metastatic	M	White or Caucasian	Non-Hispanic	72	N	0
5	metastatic	M	Black or African American	Non-Hispanic	74	Y	50
6	metastatic	M	White or Caucasian	Non-Hispanic	55	Y	0
7	metastatic	F	White or Caucasian	Non-Hispanic	73	Y	20
8	metastatic	M	White or Caucasian	Non-Hispanic	75	Y	10
9	metastatic	F	White or Caucasian	Non-Hispanic	48	N	30
10	metastatic	F	White or Caucasian	Non-Hispanic	66	Y	0
11	metastatic	M	White or Caucasian	Non-Hispanic	85	N	18
12	metastatic	F	White or Caucasian	Non-Hispanic	67	N	45
13	metastatic	F	White or Caucasian	Non-Hispanic	66	Y	0
14	metastatic	F	White or Caucasian	Non-Hispanic	62	Y	0
15	metastatic	M	White or Caucasian	Non-Hispanic	58	Y	0

Table S2: Key differentially expressed genes in pre-treatment and on-treatment CTC samples based on patient survival duration

Gene	Fold Change		Change between On-Treatment/Pre-Treatment
	Pre-Treatment	On-Treatment	
MMP9	22.98	--	↓
ALDH2	3.86	12.42	↑
ZEB2	3.43	--	↓
S100P	3.16	-2.97	↓
ADAM9	2.52	2.2	↓
MKI67	2.02	5.3	↑
MYC	-2.88	-2.38	↑
BCL2	-4.93	-4.63	↑
CTNNA1	3.95	4.02	↑

Table S3: Differentially expressed genes in thirteen overlapping deregulated pathways in pre-treatment and on-treatment samples.

Pathway	pre-treatment only		on-treatment only		both		pre-treatment only		on-treatment only		both	
	up	down	up	down	up	down	up	down	up	down	up	down
VEGFA-VEGFR2 Signaling	31	20	6	8	13	7	AP2A1, ARF4, ATP6V0D1, CAMKK2, CCRL2, CDC42BPB, EGR1, FOXO3, GAB1, LMO2, MAP2K3, MAPK1, MAPK14, MICAL2, MKNK1, PGD, PGK1, PRKCD, PTPRJ, QKI, RAC1, RAF1, SH3BGRL3, SIAH2, SLC8A1, STAT3, TAL1, TKT, TPP1, TUBA1C, TXN	ACKR3, ASCC3, CALR, DNAJB9, DSC1, EIF3H, EZR, FARSB, GIGYF2, HYOU1, LMAN1, NCL, NFATC2, PIK3R1, PRKCA, RPL10A, RPL5, RPL7, RPS6, TRPC1	ERG, GAPDH, HBD, HSPB1, LRRC59, SSR4	CREB1, FOXO1, NAP1L1, NFKB1, OCLN, PBXIP1, PRRC2C, STAT1	ADAM9, ALDOA, AP2S1, ATF4, BSG, CFL1, CTNNA1, FLII, GPX1, HDAC5, NAPA, PLA2G4A, TMOD1	APOLD1, BCL2, BIN1, ETS1, PLCG1, S1PR1, TNFRSF25
miR-targeted genes in lymphocytes	27	20	6	5	3	6	ANPEP, AP2A1, ARF4, ATP6V0A1, CORO1C, E2F3, IFRD1, MAPK14, METTL7A, MIR15A, MIR16-1, MTX1, NUCB1, PICALM, PPIF, PPP3R1, PTPRJ, RAB5C, RCOR1, RHOG, RTN4, SH3BGRL3, STX7, TM6SF1, UBE2J1, VAMP3, VTI1B	BCKDHB, CAND1, CHORDC1, GNPNAT1, GRPEL2, MATR3, MIR17, MIR20A, MSI2, MTRR, NCL, NT5E, PRIM1, RCN2, SLC25A32, SRPRB, SUCLG2, SYPL1, TAF9B, UTP15	ATP6V1F, BRI3BP, NEDD4, PPP2R4, RAB34, TYMS	KRAS, NUFIP2, PTBP2, SPCS3, SYNE1	CYP1B1, FADS2, G6PD	ANAPC1, BCL2, PDCD4, SLC38A1, SLC4A7, THEM4
miR-targeted genes in muscle cell	21	19	5	9	3	1	ANPEP, ARF4, ATP6V0A1, CORO1C, E2F3, IFRD1, LRP1, MAPK14, METTL7A, MIR15A, MIR16-1, NUCB1, PICALM, PPIF, PTPRJ, RHOG, RTN4, SH3BGRL3, STX7, TM6SF1, UBE2J1	ANAPC1, CAND1, CHORDC1, GNPNAT1, GRPEL2, MATR3, MIR17, MIR20A, MTRR, NCL, NT5E, PDCD4, PHLDB2, RCN2, SLC25A32, SLC38A1, SLC4A7, SRPRB, SYPL1	ERG, NEDD4, PPP2R4, RAB34, TYMS	ANAPC1, KRAS, NUFIP2, PDCD4, PTBP2, SLC38A1, SLC4A7, SPCS3, SYNE	CYP1B1, FADS2, G6PD	BCL2
PI3K-Akt Signaling	14	9	1	4	2	10	FLT3, FOXO3, GNB2, GNB4, HGF, IKBKG, JAK2, MAPK1, PHLPP1, PIK3AP1, PIK3CB, RAC1, RAF1, SYK	EIF4B, FASLG, FGF9, HSP90AB1, IL2RB, ITGA6, PIK3R1, PRKCA, RPS6	EIF4EBP1	CREB1, DDIT4, KRAS, NFKB1	ATF4, GNG5	BCL2, CCND2, FLT3LG, IL2RA, IL7R, ITGB7, LPAR6, MYC, RBL2, THEM4
IL-18 Signaling	9	6	2	3	4	11	CXCL16, GATA1, GSK3A, MAPK1, MAPK14, PRKCD, RAF1, STAT3, STK40	APBA2, FASLG, PARP1, PIK3R1, PRKCA, RPS6	HSPB1, PTX3	CREB1, IL18BP, NFKB1	BSG, CD36, GRN, IL18	BAZ1B, BCL2, BIN1, BIRC3, IFNG, IL2RA, LCK, LONP2, PLCG1, SLC4A7, TRAF1
MAPK Signaling	15	8	2	3	2	5	DUSP3, IKBKG, MAP2K3, MAPK1, MAPK14, MKNK1, PPM1A, PPP3R1, PRKCD, RAC1, RAF1, RAPGEF2, RASGRP4, STK3, ZAK	CACNA1I, FASLG, FGF9, HSPA8, MAP3K4, PPP3CC, PRKCA, RASGRF2	HSPB1, RRAS	KRAS, MAP4K1, NFKB1	ATF4, PLA2G4A'	CDC25B, DUSP16, ELK4, MYC, RASGRP
Nuclear Receptors	18	3	4	4	5	4	ABCC3, CPEB4, EGR1, FGD4, FTL, GSR, HGF, PGD, RXRA, S100P, SEC14L1, SLC6A6, SLC6A8, SRGN, STAT3, TSC22D3, TXN, VDR	HSP90AB1, MFGE8, SMC1A	GCLM, MGST3, PPP2R4, SLC2A13	BHLHE40, FOXO1, POU5F1, PPARD	BLVRB, CYP1B1, G6PD, MGST1, MGST2	BIRC3, IFNG, MYC, TGFBR3
Ciliary Landscape	7	16	1	5	3	2	AGPAT2, EXOC6, EXOC7, MKLN1, RAC1, RMND5A, WDR26	AIMP1, BBS9, CALM1, CLUAP1, EXOC6B, EXOSC2, EXOSC7, EXOSC9, MCM3, MCM7, MCM8, MSH2, NUP88, PGRMC2, TBC1D4, UQCC1	MCM4	APC, CNOT1, LRPPRC, NFKB1, PSMC6	GLA, LCN2, MYL6	CEP290, RNGTT
Ras Signaling	12	6	1	2	3	5	ETS2, FLT3, GAB1, GNB2, GNB4, IKBKG, MAPK1, PIK3CB, RAB5C, RAC1, RAF1, RASGRP4	CALM1, FASLG, PIK3R1, PRKCA, RASGRF2, ZAP70	RRAS	KRAS, NFKB1	CALM2, GNG5, PLA2G4A	ETS1, PLCG1, RASGRP1, SYNGAP1, TIAM1
T-Cell antigen Receptor (TCR) Signaling	6	10	1	3	--	9	IKBKG, MAPK1, MAPK14, PRKCD, RAF1, VAV3	CD247, CD3D, CD8A, FYN, GATA3, MALT1, NFATC2, PIK3R1, PRKCQ, ZAP70	DBNL	CREB1, MAP4K1, NFKB1	--	BLB, CD28, CD3E, CD3G, ICOS, ITK, LCK, PLCG1, SKAP1
CAMKK2	12	7	3	2	1	5	CAMKK2, CTSE, CYB5R3, ERMAP, FAM20C, HK2, MAP1LC3B, MAPK1, NEDD4L, RAC1, S100A8, TMEM176B	CALM1, NFATC2, PARP1, PLAC8, PRKCA, RPS6, SFI1	EIF4EBP1, HMBS, RHAG	CREB1, SELL	HK1	CAMK4, CCND2, CD28, MAF, RASGRP1
Focal Adhesion-PI3K-Akt-mTOR-signaling	10	7	1	4	2	5	FOXO3, GNB2, GNB4, HGF, IKBKG, JAK2, MAPK1, PHLPP1, PIK3CB, RAF1	EIF4B, FGF9, HSP90AB1, IL2RB, ITGA6, PIK3R1, RPS6	EIF4EBP1	CREB1, DDIT4, FOXO1, KRAS	ATF4, GNG5	IL2RA, IL7R, ITGB7, LPAR6, PIK3IP1
EGF/EGFR Signaling	12	3	2	6	4	3	AP2A1, ASAP1, GAB1, JAK2, MAPK1, MAPK14, PRKCD, RAC1, RAF1, REPS2, STAT3, VAV3	MAP3K4, PIK3R1, PRKCA	EIF4EBP1, NEDD4	CREB1, FOXO1, KRAS, MAP4K1, STAT1, STAT5B	AP2M1, AP2S1, AURKA, CFL1	CBLB, ELK4, PLCG1
